# Sequencing of Chloroplast Genomes from Wheat, Barley, Rye and Their Relatives Provides a Detailed Insight into the Evolution of the Triticeae Tribe

**DOI:** 10.1371/journal.pone.0085761

**Published:** 2014-03-10

**Authors:** Christopher P. Middleton, Natacha Senerchia, Nils Stein, Eduard D. Akhunov, Beat Keller, Thomas Wicker, Benjamin Kilian

**Affiliations:** 1 Institute of Plant Biology, University of Zurich, Zurich, Switzerland; 2 Laboratory of Evolutionary Botany, Institute of Biology, University of Neuchâtel, Switzerland; 3 Leibniz-Institute of Plant Genetics and Crop Plant Research, Gatersleben, Germany; 4 Department of Plant Pathology, Kansas State University, Manhattan, Kansas, United States of America; KU Leuven, Belgium

## Abstract

Using Roche/454 technology, we sequenced the chloroplast genomes of 12 Triticeae species, including bread wheat, barley and rye, as well as the diploid progenitors and relatives of bread wheat *Triticum urartu*, *Aegilops speltoides* and *Ae. tauschii*. Two wild tetraploid taxa, *Ae. cylindrica* and *Ae. geniculata*, were also included. Additionally, we incorporated wild Einkorn wheat *Triticum boeoticum* and its domesticated form *T. monococcum* and two *Hordeum spontaneum* (wild barley) genotypes. Chloroplast genomes were used for overall sequence comparison, phylogenetic analysis and dating of divergence times. We estimate that barley diverged from rye and wheat approximately 8–9 million years ago (MYA). The genome donors of hexaploid wheat diverged between 2.1–2.9 MYA, while rye diverged from *Triticum aestivum* approximately 3–4 MYA, more recently than previously estimated. Interestingly, the A genome taxa *T. boeoticum* and *T. urartu* were estimated to have diverged approximately 570,000 years ago. As these two have a reproductive barrier, the divergence time estimate also provides an upper limit for the time required for the formation of a species boundary between the two. Furthermore, we conclusively show that the chloroplast genome of hexaploid wheat was contributed by the B genome donor and that this unknown species diverged from *Ae. speltoides* about 980,000 years ago. Additionally, sequence alignments identified a translocation of a chloroplast segment to the nuclear genome which is specific to the rye/wheat lineage. We propose the presented phylogeny and divergence time estimates as a reference framework for future studies on Triticeae.

## Introduction

The tribe of Triticeae is within the subfamily of the *Pooideae* and comprises between 400–500 species including diploids and polyploids. It includes several major crop species such as, *Hordeum vulgare* (barley), *Secale cereale* (rye) and *Triticum aestivum* (wheat). These species have undergone many changes during the domestication process, with the domesticated taxa being distinct from their wild ancestors [1], [2]. However, it was through domestication that these taxa became important for agriculture, with wheat becoming one of the most important crop species.

Triticeae include many polyploid species. The most important is bread wheat (*Triticum aestivum*), an allohexaploid, which has three genomes (A, B, and D) and an approximate genome size of 16–17 Gb [3], [4]. The haploid genome sizes of the progenitors are similar to the haploid genome sizes of other Triticeae and are generally between 3,5–8,5 Gb [5]–[7]. The complete genome complement of *T. aestivum* was formed from the hybridisation of three diploid ancestors. The first hybridisation event was estimated to have occurred 0.20 to 1.3 million years ago, between *T. urartu* (AA) and a yet unidentified B genome species to form the tetraploid *T. dicoccoides* [8], [9]. The exact origin of the B genome is still unclear, but a closely related species or an ancestral relative of *Ae. speltoides* (S genome) has been suggested to be the likely donor [8], [9]. The D genome was added to the domesticated tetraploid *T. dicoccon* from *Ae. tauschii* approximately 8,000–10,000 years ago to form the complete hexaploid genome complement of *T. aestivum* [10]–[13].

Other polyploids within the Triticeae include *Aegilops cylindrica* (jointed goatgrass), a tetraploid containing the C and D genomes and *Aegilops geniculata* (ovate goatgrass) which comprises the M and U genomes [14]. The exact phylogenetic relationships of the C, M and U genomes with others (e.g. the A, B and D genomes) are less than clear. PCR fragment polymorphism analyses have generally placed the U and M genome closer to D than to the A and B genomes [15], [16]. Furthermore, U and M are probably more closely related to the D genome than C [15]. In the case of *A. cylindrica*, there is evidence for multiple polyploidisation events, with both the C and the D genome donor acting as maternal parent [17]. *A. cylindrica* is of worldwide economic importance as a weed of bread wheat.

Of less agricultural importance is *T. monococcum* (Einkorn wheat), which contains the A genome and was domesticated from its wild progenitor *T. boeoticum*. Both of these taxa are closely related to *T. urartu*, the A genome donor of *T. aestivum* [18], [19]. Even though *T. urartu* and *T. boeoticum* are closely related, they can be crossed, but produce sterile hybrids, indicating that their phylogenetic distance is large enough to form a species boundary [18]. *H. vulgare* is another agriculturally important species and was domesticated from its wild progenitor *H. spontaneum*.

The evolution and the divergence times of several species of Gramineae have been studied: *Sorghum bicolor* (sorghum), diverged approximately 60 million years ago (MYA) [20], [21], *Oryza sativa* (rice) approximately 40–53 MYA, and *Brachypodium distachyon* diverged approximately 32–39 MYA from the Triticeae [22], [23]. Within Triticeae inferred dates of divergence are sometimes vague: *H. vulgare* is estimated to have diverged from rye and wheat 10–15 MYA . *S. cereale* and wheat diverged approximately 5–11 MYA, and the ancestral genome donors *T. urartu*, *Ae. speltoides* and *Ae. tauschii* were estimated to have diverged from each other between 2 and 6 MYA [24]–[26]. It is important to narrow down these estimates to gain a better understanding of the evolution of the Triticeae. This is particularly the case for the divergence times of *T. urartu*, *Ae. speltoides* and *Ae. tauschii*, which have received little attention, as focus on these species has generally been in their contribution to the *T. aestivum* genome.

The size of the chloroplast genome is usually between 115 and 165 Kb [27]. The composition of chloroplasts from the Poaceae family is very similar between species and consists of a large single copy region (LSC), which is approximately 80 kb, and a small single copy region (SSC) of approximately 13 kb in length, located between the two inverted repeat sequences of approximately 20 kb [28], [29].

To date the sequences of approximately 230 chloroplast genomes are publically available. Some of these, including *H. vulgare* and *S. bicolor*, have been used in comparative analysis to ascertain phylogenetic relationships between grasses [29], while Chaw *et al*,. 2004, used whole chloroplast sequences from twelve taxa to date the divergence time between eudicots and monocots to 140–150 MYA. In addition, Nikiforova *et al*,. 2013 used complete chloroplast sequences from 47 apple species, including wild and domesticated species to date the divergence times of the individual species. Advantages of using chloroplasts are that they are non-recombining and haploid and can be treated as a single locus and that they are maternally inherited [30], [31].

The origin of the *T. aestivum* chloroplast genome has been investigated in several studies [30], [32]. Golovnina *et al*,. 2007 used the chloroplast *matk* gene along with the *trn*L intron sequence from a large number of Triticeae species and found that the *Ae. speltoides* chloroplast genome sequence had the highest similarity to the chloroplast genome sequence of *T. aestivum*. Therefore, they suggested that *Ae. speltoides* was a close relative of the diploid species that donated its chloroplast genome to *T. aestivum*.

Previous approaches to establishing phylogenetic relationships have focused on sequencing one or a small sample of genes [33]. However, with the rise of high throughput sequencing which provides much larger datasets for each of the species, chloroplast genomes can usually be assembled as a side product of survey sequencing projects [31].

Here we used Roche/454 sequencing to obtain chloroplast sequences for 12 Triticeae species with a coverage of between approximately 22× and 92×. These included the three A, B and D genome donors of *T. aestivum*. We wanted to address the following questions: (i) What are the divergence times of the species studied? (ii) Which of the sub-genome donors contributed their chloroplasts to the polyploids such as *T. aestivum*, *A. cylindrica* and *A. geniculata* ? (iii) How do the chloroplast genomes of individual Triticeae species differ at the DNA level? We estimated the divergence times of of all 12 Triticeae species from each other and found the times to be more recent than previous estimates. Additionally, a close relative of *Ae. speltoides* was confirmed as the chloroplast donor of *T. aestivum* and a relative of *Ae. tauschii* was identified as the possible chloroplast donor to *A. cylindrica*.

## Results

### Chloroplast genome assemblies

A single run of 454 titanium 7 kb paired end sequencing was conducted on genomic DNA of 11 Triticeae species and subspecies (Table 1) and additional sequences for *T. aestivum* were provided by the University of Bristol. We included two domesticated taxa *H. vulgare* ssp. *vulgare* and *T. monococcum* ssp. *monococcum* and their wild subspecies progenitors *H. vulgare* ssp. *spontaneum* and *T. monococcum* ssp. *boeoticum*. From here on in the text these subspecies will be referred to as *H. spontaneum* and *T. boeoticum* respectively.

**Table 1:** Chloroplast assembly information for all twelve Triticeae taxa.

Species name^a^	Total reads	Cp reads^b^	Cp reads[%]^c^	Coverage	Cp size[bp]^d^
*T. aestivum* (Chinese Spring)	499,999	7,972	1.59	25	135,509
*T. urartu* (EP0471)	546,057	10,825	1.98	22	135,945
*Ae. speltoides* (SPE0061)	441,540	10,934	2.48	24	134,865
*Ae. tauschii* (AE429)	640,266	12,564	1.96	26	134,268
*Ae. cylindrica* (TA2204)	667,485	26,672	4.00	35	133,444
*Ae. geniculata* (TA1800)	646,327	25,842	4.00	32	137,231
*T. boeoticum* (1628)	458,875	16,868	3.68	40	134,928
*T. monococcum* (2240)	507,523	13,093	2.58	37	136,923
*S. cereale* (Imperial)	586,127	18,783	3.20	43	135,604
*H. vulgare* (Barke)	1325,384	20,133	1.52	86	135,802
*H. spontaneum* (FT11)	659,263	28,145	4.27	87	135,549
*H. spontaneum* (FT462)	642,312	31,158	4.85	92	135,864

a Taxon name, cultivar or accession names are given in parentheses.

b Number of 454 of reads mapping on the chloroplast.

c Proportion of 454 reads mapping on the chloroplast.

d Chloroplast size: including the second inverted repeat.

**Figure 1:**
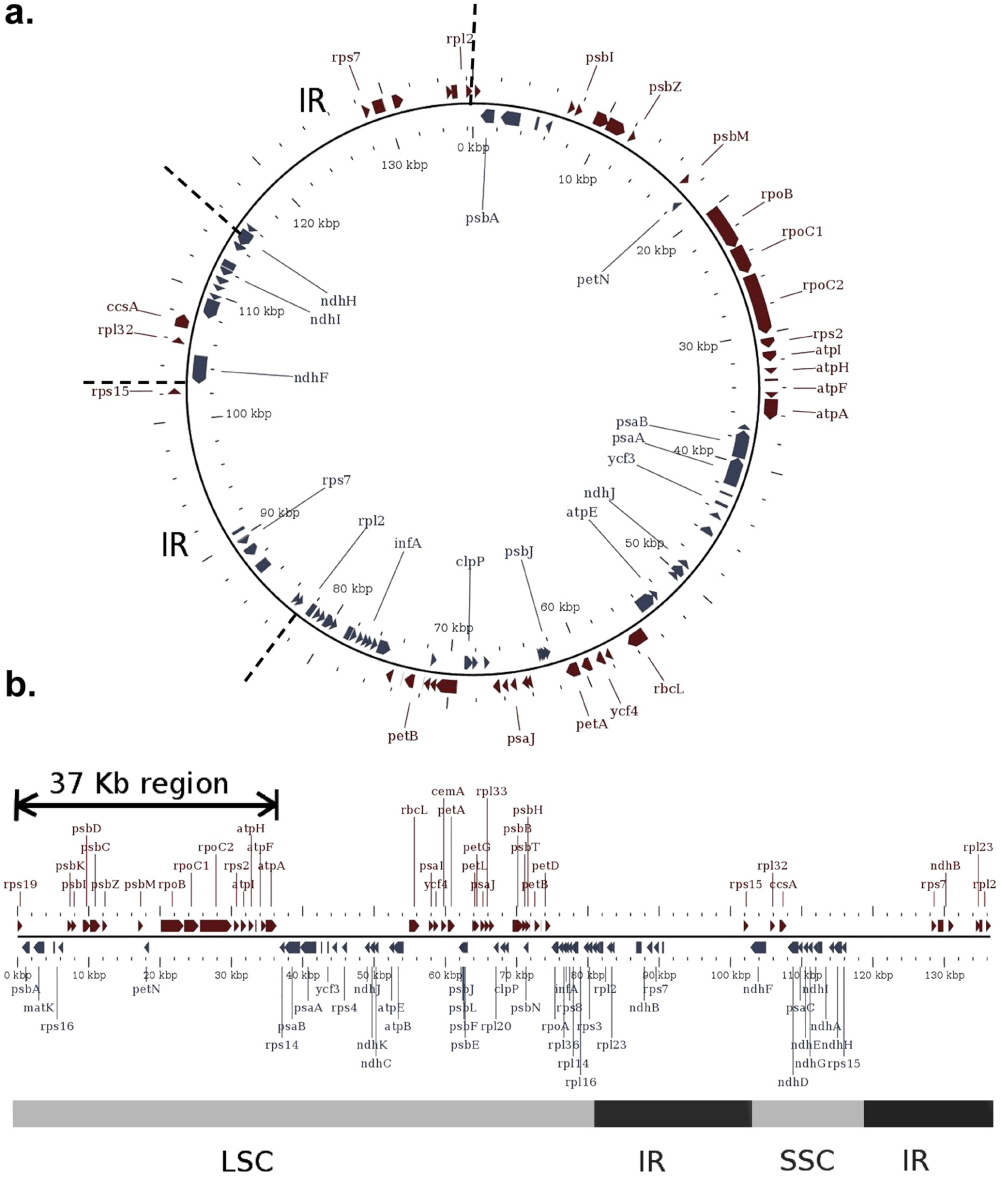
Map of the barley (*H. vulgare*) chloroplast that was used as a reference for the assembly of other Triticeae chloroplast sequences. **a.** Diagram of the layout of the *H. vulgare* chloroplast genome showing the LSC (large single copy, ∼80 kb), the SSC (small single copy, ∼8 kb) and the two inverted repeat (IR) sequences (∼20 kb each). **b.** Shows the sequence assembly, with the arrow representing the 37 kb region chosen for analyses of divergence times.

As organellar DNA was not excluded in the DNA extraction, between 1.96% and 4.27% of the total number of reads for each taxon originated from the chloroplast (Table 1). Chloroplast DNA insertions into the nuclear DNA make up less than 0.01% of genomic DNA [34]–[36], this equates to approximately 50 reads per run. We therefore conclude that it is unlikely that these reads are interfering with the chloroplast sequence assemblies. Due to the relatively small size of the chloroplast genome (≈150 kb), the large number of reads gave a high coverage for each of the chloroplast genomes of 20 to 90 fold (Table 1). This allowed for high quality assemblies of all of the chloroplast genomes because at such high sequence coverage, the number of sequencing errors in the final assembly is negligible [37]. However, the assembly was hampered by the inverted repeat sequence (IR), which could not be resolved into two separate copies with the available sequences and would have required specific and time consuming laboratory procedures. Thus only single-copy regions and one unit of the IR are contained in our chloroplast genome assemblies (Figure 1).

### Origin of chloroplasts in polyploid Triticeae species

We first wanted to study if it was possible to conclusively establish the origin of the *T. aestivum* chloroplast either from *T. urartu*, *Ae. speltoides* or *Ae. tauschii*, the A, B, and D genome donors respectively. A 37 Kb sequence from the large single-copy region was chosen for the alignment, because this region is highly conserved between all grass species (allowing reliable sequence alignments) and is not part of the inverted repeat sequence (Figure 1). This region begins in the intergenic sequence 77 bp upstream from the start of the photosystem II protein D1 coding gene (*psbA*) and ends 5 bp before the start of the photosystem I P700 chlorophyll A apoprotein A2 coding gene (*psaB*), (Figure 1).

**Figure 2:**
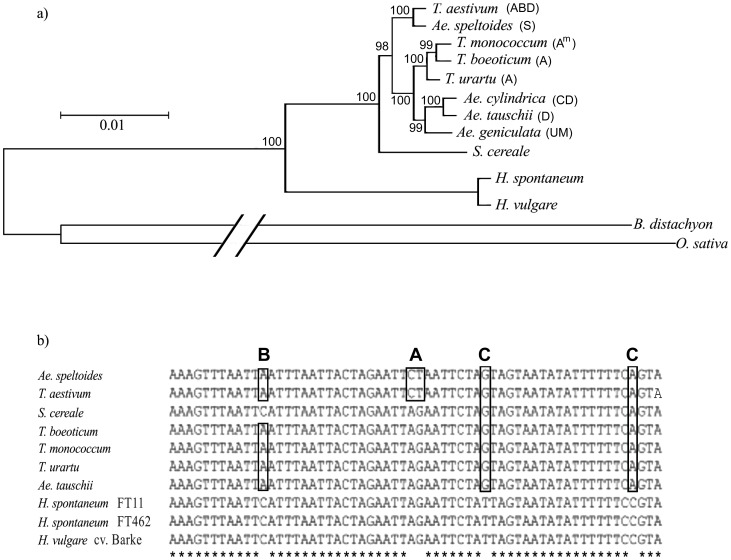
Comparison of Triticeae chloroplast sequences. **a.** Maximum likelihood tree for chloroplast sequences drawn with MEGA 5.0 [56] using 1,000 bootstrap repetitions with *B. distachyon* and *O. sativa* as as outgroups. The genome formula for wheat and its close relatives is indicated in parentheses. It shows that *Ae. speltoides* has the highest sequence similarity to the chloroplast of *T. aestivum*. This indicates that *Ae. speltoides* or a close relative is the chloroplast donor for *T. aestivum*. **b.** Examples of diagnostic polymorphisms. The outlined sequences in (A) are those that are identical between *Ae. speltoides* and *T. aestivum*. (B) indicates nucleotides that are identical among the wheats, but different in the other sequences, and (C) indicates positions that distinguish barley from all others.

All 12 taxa were used in multiple sequence alignments of the 37 kb region and 1,000 bootstrap replications were used to draw the phylogenetic tree, using the previously published *B. distachyon* and *O. sativa* chloroplast genome sequences as outgroups (Figure 2). The chloroplasts from the three A genome taxa *T. urartu*, *T. boeoticum* and *T. monococcum* are closely linked in the tree along with the chloroplast from the D genome taxon *Ae. tauschii*. However, it is the chloroplast from *Ae. speltoides* that shows the closest phylogenetic relationship with the chloroplast from *T. aestivum* (Figure 2). Pairwise analysis of the 37 Kb region was used to determine the sequence similarity between the taxa. The chloroplast sequences of *T. aestivum* and *Ae. speltoides* were found to be 99.87% identical (i.e only 11 polymorphisms in the 37 kb sequence). The other chloroplast genome sequences of the wheat genome donors *T. urartu* and *Ae. tauschii* showed lower sequence identity to *T. aestivum*, ranging from 99.64%–99.69% and *T. urartu* was found to have a greatest similarity to *Ae. tauschii*, with 99.76%. Additionally, multiple alignments showed many polymorphisms that are only found in the sequences of *T. aestivum* and *Ae. speltoides* that clearly grouped the *T. aestivum* and *Ae. speltoides* chloroplast sequences together (examples are given in figure 2b). Therefore, we conclude that the donor of the *T. aestivum* chloroplast was closely related (but not identical to) today's *Ae. speltoides*.

The chloroplast sequences from the two tetraploid species *Ae. geniculata* and *Ae. cylindrica* were were clustering with chloroplast sequence of *Ae. tauschii* (D genome, Figure 2a). Because the C genome of *Ae. cylindrica* was described to be more distant from D than both U and M [15], our data indicate that the chloroplast of *Ae. cylindrica* was donated by the D genome parent.

### Evidence for the migration of a chloroplast sequence to the nuclear genome

The complete chloroplast genome sequences of *H. vulgare* and *T. aestivum* were directly compared at the sequence level. We identified four deletions and five insertions (InDels) greater than 50 bp in the chloroplast genome sequence of *T. aestivum*, compared to the chloroplast genome sequence of *H. vulgare*.

**Figure 3:**
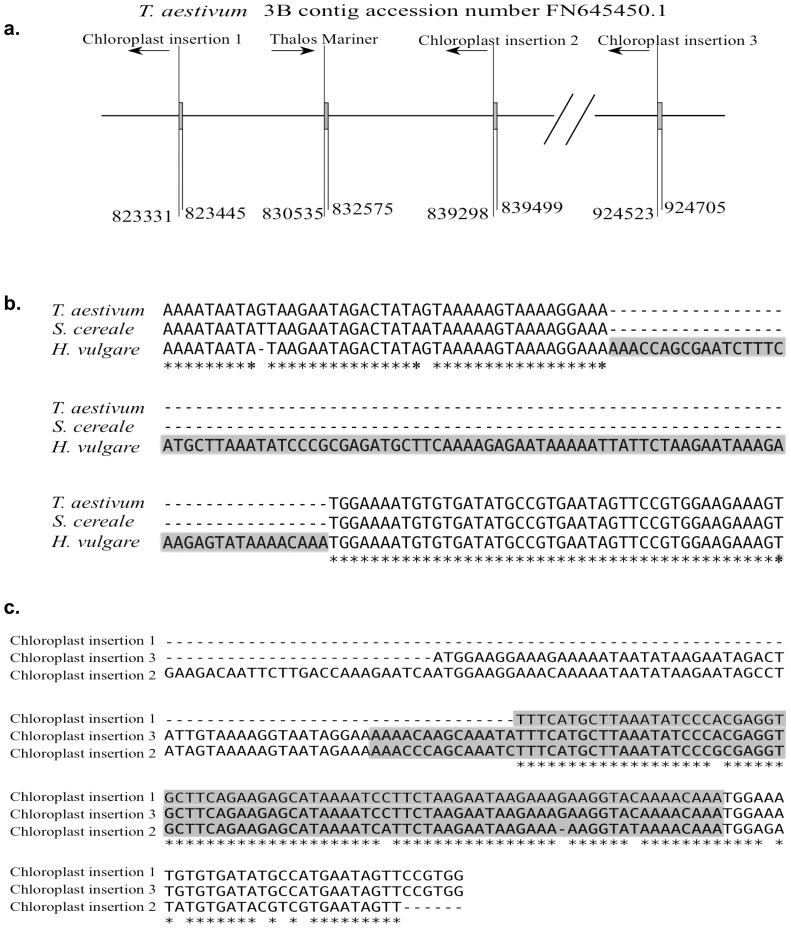
Putative migration of a chloroplast segment into the nuclear genome in the wheat/rye lineage. **a.** Chromosome 3B section containing a duplicated chloroplast insertion. **b.** A region of the alignment of the chloroplast sequences of *H. vulgare* and *T. aestivum*, showing the removed sequence from the *T. aestivum* chloroplast genome sequence. **c.** Alignment of the three identified chloroplast sequences from chromosome 3B of *T. aestivum* showing that this sequence has undergone a possible duplication event after excision from the chloroplast genome. The highlighted region shows the absent region in the *T. aestivum* chloroplast genome (**b.**).

One sequence of 92 bp is present in the *H. vulgare* chloroplast sequence (position 17,126–17,218 bp), but is absent in the same position of the *T. aestivum* chloroplast genome sequence. Interestingly, a homologue of this small region was found on a sequence contig from *T. aestivum* chromosome 3B (accession number FN645450.1). It was found in three locations along the contig in positions 823,371–823,445 (74 bp), 839,298–839,499 (201 bp) and 924,523–924,705 (182 bp), with the sequence from the *H. vulgare* chloroplast being 89% identical to the sequences found on chromosome 3B in *T. aestivum*. Alignments of the three chloroplast sequences revealed that they have been duplicated after insertion into the nuclear genome (Figure 3). We propose that a chloroplast genome segment was copied from the chloroplast genome and inserted into the nuclear genome on chromosome 3B. The segment was then duplicated on chromosome 3B and subsequently degraded from the chloroplast organellar genome sequence.

All other chloroplast genomes were searched for this deleted sequence, and it was only found in the chloroplast genome sequences of *H. vulgare* and the two wild *H. spontaneum* genotypes. This indicates that this region moved from the chloroplast to the nuclear genome in the lineage leading to rye and wheat after the divergence from barley (Figure 3).

### Triticeae divergence time estimates based on chloroplast sequences

**Figure 4:**
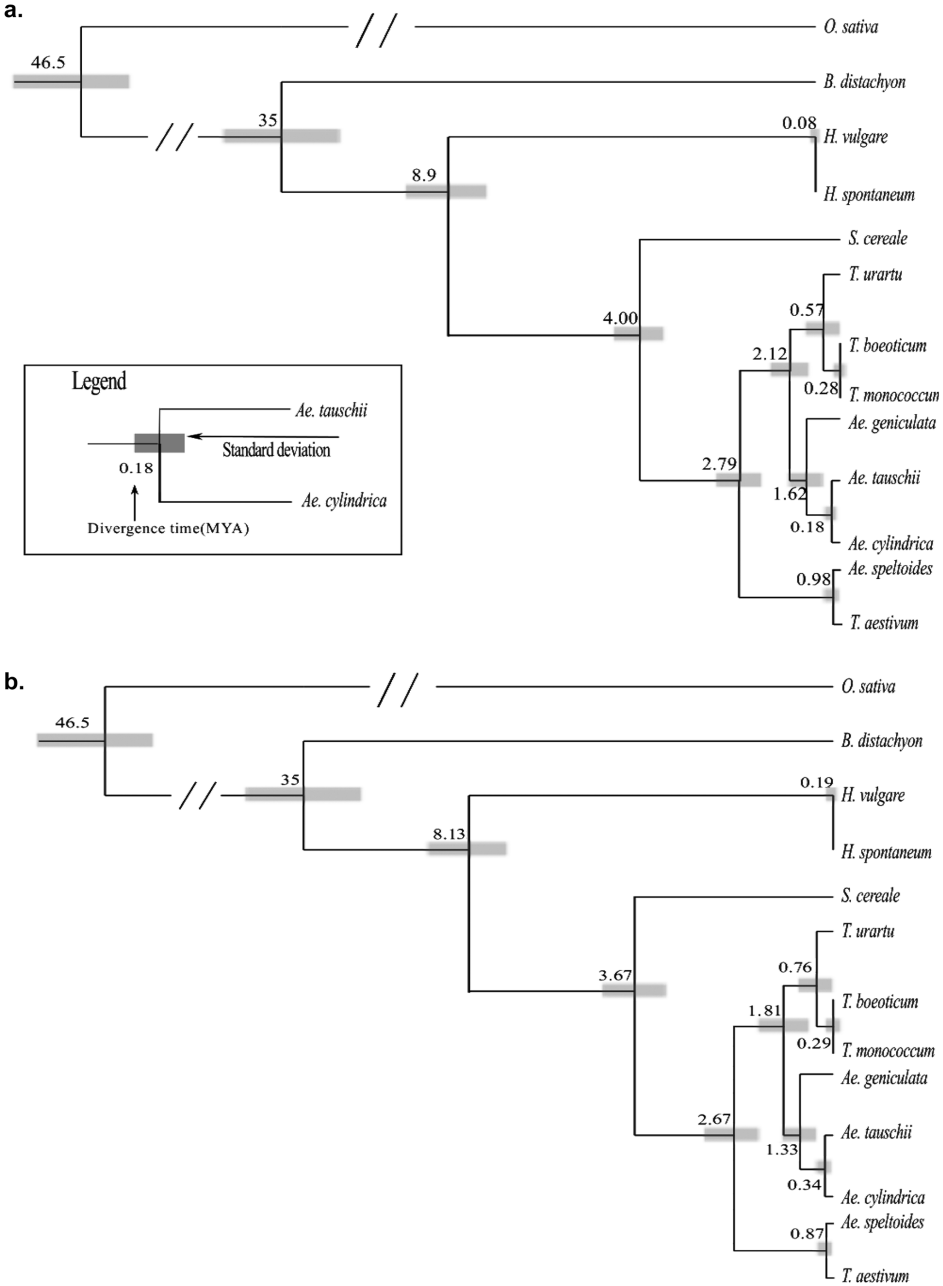
Divergence time estimates of Triticeae species based on chloroplast sequences. **a.** Divergence times of the species based on the strict clock method, using a substitution rate of 1.06E-3per base per million years calculated using PAUP and r8s programs over the selected 37 kb region of the chloroplast genome. Divergence times are represented in million years. **b.** Divergence time estimates based on an uncorrelated relaxed clock method. Both trees were drawn using the divergence of *O. sativa* and *B. distachyon* as anchor points. The legend describes the divergence time in million years and the grey boxes represent the standard deviation of the divergence times. The precise numbers for standard deviations are given in Table 2.

Phylogeny of the Triticeae species used in this study was drawn from the chloroplast sequences. Both a maximum likelihood and Bayesian methods were used to obtain divergence time estimates of these species. The maximum likelihood tree was generated using MEGA 5.0, with 1000 bootstrap replicates and a GTR+G+I model of substitution. A topologically identical tree was produced using MrBayes under the same substitution model (Figure 4). To estimate divergence times, we used the 37 Kb chloroplast region described above. This region contains 21 genes, 15 tRNAs and seven intergenic sequences greater than 1 kb.

Two distinct methods were also employed to test the validity of the estimates of divergence of the Triticeae used in this project. The first method used a penalized likelihood method assuming a strict molecular clock. Several programs were implemented in order to obtain estimates for the divergence times of the Triticeae species. These included PAUP* (Swafford, 2002) for phylogenetic analysis and tree building, jModeltest [38] to identify the best substitution model of mutation rates as a basis for age estimates and r8s [39] to infer divergence times and to test the consistency of the molecular clock along the branches of the phylogenetic tree. We used the previous estimate that *B. distachyon* and *O. sativa* diverged from *T. aestivum* approximately 32–38 MYA and 40–53 MYA respectively [23], as anchor points for the calculations of the divergence times. Based on the divergence of *O. sativa* and *B. distachyon*, the overall substitution rate for the 37 kb region was calculated to be 1.06E-3 per base per million years. From this the divergence time of *H. vulgare* from *T. aestivum* was found to be 8.9 MYA±0.9, (sequence identity of 97.84%). *S. cereale* was found to have diverged from *T. aestivum* 4.0MYA±0.5 (sequence identity of 99.43%). Both of these estimates of divergence times are more recent and have a smaller deviation than previously published estimates [24]–[26]. The divergence time of the tetraploid *Ae. geniculata* from D genome species was found to be 1.62±1 MYA and *Ae. cylindrica* diverged from *Ae. tauschii* approximately 0.18±0.05 MYA (Figure 4a).

The second method to estimate the divergence times used Bayesian inference as implemented in the software BEAST [40]. The dates of divergence where also based on the calibration points from *O. sativa* and *B. distachyon* described above. The divergence times were calculated twice, using a fixed and an uncorrelated relaxed molecular clock. The results of the relaxed clock analysis are shown in Figure 4b while the comparison of relaxed and strict clock analyses is shown in Table 2. Because the relaxed clock allows for different substitution rates in different branches of the tree, the estimates of divergence times have larger confidence intervals than with the strict clock (Table 2). In general, the divergence times derived from the Bayesian approach were very similar to those using a penalized likelihood approach with the Bayesian estimates being slightly more recent (Figure 4). The barely/wheat divergence was placed at 8.13±2.13 MYA while the divergence of *S. cereale* from wheat was 3.67±1.5 MYA. The split into the three clades containing the A, B and D genome donors occurred 2.67±1.1 MYA. A further split approximately 1.81±0.8 MYA resulted in the clades containing the A and D genome taxa including *Ae. geniculata*. The divergence of *Ae. speltoides* from *T. aestivum* was estimated to have occurred 0.87±0.5 MYA (Figure 4b). In addition to using the whole 37 kb interval, we also generated partitions using genes and intergenic sequences separately. The results were virtually identical to those where the sequence was used as a whole.

**Table 2:** Divergence time estimates used Bayesian inference, applying strict and relaxed molecular clocks.

Node^a^	Divergence time^b^	STD^c^	Divergence time^d^	STD^e^
*H. vulgare/H. spontaneum*	0.19	0.17	0.09	0.06
*H. vulgare/T. aestivum*	8.13	2.13	8.19	0.73
*S. cereale/T. aestivum*	3.67	1.46	3.13	0.39
B genome/A+D genomes	2.67	1.10	2.21	0.30
D genome/A genome	1.81	0.79	1.39	0.21
*Ae. tauschii/Ae. geniculata*	1.33	0.62	1.16	0.20
*Ae. tauschii/Ae. cylindrica*	0.34	0.25	0.19	0.09
*T. urartu/T. boeoticum*	0.76	0.45	0.55	0.15
*T. boeoticum/T. monococcum*	0.29	0.22	0.29	0.10
*T. aestivum/Ae. speltoides*	0.87	0.49	0.78	0.20

a Node of the phylogenetic tree, see also Figure 4.

b Divergence time estimate in million years, using a relaxed clock.

c Standard deviation.

d Divergence time estimate in million years, using a strict clock.

### Divergence time calculations of the subspecies

It was also possible to date the divergence of *T. boeoticum* from *T. urartu* to approximately 0.57±0.14 MYA using the penalized strict clock method and 0.76±0.45, using the relaxed clock method. The divergence time between *T. boeoticum* and *T. monococcum* could not be calculated using the semi penalized likelihood estimation. However, this was resolved using Bayesian inference, with the estimated time of divergence being 0.29±0.22 MYA (using a relaxed clock) and 0.29±0.2 MYA (using a strict clock, Table 2).

*H. vulgare* and *H. spontaneum* are very closely related (sequence identity of 99.98%). The divergence time of the two was calculated to be 80,000±20,000 years using semi penalized likelihood. Using the Bayesian approach with a strict clock yielded a very similar number of 90,000±60,000 years and approximately double this with a relaxed clock, 190,000±170,000 MYA.

## Discussion

The objectives of this study were to examine the relationships of Triticeae species using chloroplast genome sequences. Because the 454 titanium sequencing generated on average 500,000 reads from genomic DNA per taxa, it resulted in approximately 8,000–30,000 reads that were derived from the chloroplast. This led to high quality assemblies of the chloroplast genomes from all taxa, and allowed us to calculate the divergence times of several Triticeae taxa and draw conclusions on the origin of chloroplasts in polyploid species.

### Barley, rye and wheat diverged within the past 8–9 million years

The central aim of our study was to obtain more precise estimates for the divergence times of Triticeae species. For these estimates, we used a 37 kb segment of chloroplast sequence which contained both protein-coding and non-coding regions. Most previous divergence time estimates were based on intergenic sequences or synonymous sites of coding regions [9], [24], [26]. We argue that it is legitimate to use large segments that contain both intergenic and genic sequences. We partitioned the sequence into coding and non-coding datasets. The results were virtually identical to those obtained by using the 37 Kb sequence as a whole. Furthermore, using strict clock and relaxed clock methods led to almost identical results on the partitioned and non-partitioned datasets.

Our divergence time estimates are generally more recent than estimates from previous studies [9], [24], [26]. However, due to limited availability of genomic sequences, previous estimates were based on single or very few gene sequences which consequently lead to relatively large standard deviations for the estimates. In particular the divergence of *H. vulgare* from wheat which was estimated previously to have occurred approximately 8–12 MYA [9], [24], [26] is shifted to more recent 8.1 and 8.9 MYA using strict and relaxed clock methods, respectively. These values are still in the range of estimates reported by Chalupska *et al.* (approx. 7–16 MYA) Dvorak *et al.* [9] (8.3–11.3 MYA) but clearly more recent than the range of 11.4±0.6 reported by Huang *et al*,. [24].

Similarly, our estimate of 3.5–4 MYA for the divergence of rye from wheat and its relatives is more recent than previous ones [24], [26]. Again, this value is still in the range of estimates reported by Chalupska *et al.* 2008 [26] but more recent than the values reported by Huang *et al.* 2002 [24] which were 3–9 MYA and 7.4±0.9 MYA, respectively.

### Phylogeny and divergence of wheat and its genome donors

Of particular interest to us was the precise phylogeny and dating of the age of the wheat genome donors. Our analysis indicates that the donors (or their close relatives) of the wheat genomes diverged within the past 3 million years. According to our phylogenetic analysis *Ae. speltoides* branched off first, followed relatively soon by the divergence of *Ae. tauschii* and *T. urartu*. Our estimates are in the range of previous ones [9], [26] which ranged rather widely from 2–6 MYA. However, we have narrowed this range of divergence to 1.9–2.5 (using a fixed clock) and 1.5–3.7 MYA (using a relaxed clock).

The precise phylogenetic relationships between the A, B and D genomes are still a topic of debate. Depending on which and how many gene loci were studied, the A, B or D genome were each once found to be the most divergent of the three [41], [42]. Reticulate evolution (i.e. hybridisation of closely related species) and incomplete lineage sorting in large populations were proposed as possible explanation for these contradictory results [42].

We also consider it possible that, due to the relatively small datasets, ancient paralogs were compared instead of true orthologs. Furthermore, several studies showed that genomic sequences of Triticeae species are composed of haplotype segments that are older than the species that were compared [43]–[45]. Thus, we must emphasize that our data only provides the phylogenetic relationships of the chloroplast lineages. This is a limitation of our approach, as new chloroplast lineages may have been introgressed independently of the actual species divergence. A general conclusion will probably only be possible once large portions of the A, B and D genomes are compared.

### Formation of a species boundary within the last 550,000–760,000 years in A genome species

Dating of three diploid Triticeae taxa containing the A genome was also conducted, these included *T. urartu*, *T. boeoticum* and *T. monococcum*. The three taxa were all found to have diverged relatively recently, with *T. urartu* diverging from the other two roughly 550,000–760,000 years ago (depending on which estimate is used, see Figure 4 and Table 2). The close relationship between *T. urartu* and *T. boeoticum* is of particular interest because it may give some indication for the time it takes to evolve a species boundary in the Triticeae tribe: these two taxa are not interfertile [18], indicating that a species boundary evolved in less than 550,000–760,000 years. Here, it has to be noted that this estimate refers to the divergence of the chloroplast lineages. Thus, the actual species divergence could be even more recent (discussed below).

In contrast, successful crosses can be made between the very closely related *T. boeoticum* and *T. monococcum* [19], indicating that both taxa are fully interfertile. Indeed, it was proposed based on archeobotanical findings that *T. monococcum* (the domesticated form of wild einkorn wheat) originated only within the last 12,000 years [19]. However, we have dated the chloroplast divergence between the two to about 280,000–290,000 years. This discrepancy can be explained by the intrinsic characteristics of molecular dating (see discussion below).

### Chloroplasts of cultivated and wild barley are very closely related

Comparison of the *H. spontaneum* (FT11) chloroplast sequence with *H. vulgare* cv. Barke showed that these two sequences are virtually identical with a sequence homology of 99.98%. The polymorphisms were distributed more or less evenly, so we excluded the possibility that one single event could be responsible for the difference (e.g. a micro-rearrangement that affected a dozen or so bp). This high level of sequence similarity translates into a divergence time of approximately 80,000±20,000 years under the strict clock assumption and approximately twice this, 190,000±170,000 years using a relaxed clock approach. This estimate was based on the *H. spontaneum* FT11 genotype from Israel. A second *H. spontaneum* genotype was also included in the analysis (FT462 from Turkey). This was found to have a virtually identical sequence to the FT11 genotype (99.98%). From this data we cannot infer the origin of *H. vulgare* cv. Barke from either of the two *H. spontaneum* accessions, as the relationship between these accessions is too close. Nevertheless, we can state that chloroplasts from cultivated and wild barley are clearly more closely related than those of other pairs of wild and domesticated Triticeae subspecies (e.g. *T. boeoticum* and *T. monococcum*, see above). The fact that our estimates for the divergence of wild and cultivated barley predate the beginning of agriculture approximately 10,000 years ago may also be explained by the characteristics of molecular dating (see discussion below).

### The *T. aestivum* chloroplast diverged from that of *Ae. speltoides* less than 1 million years ago

The high quality assemblies allowed us to conclusively determine the origin of the *T. aestivum* chloroplast genome donor. Previous studies [12], [46], [47], suggested a link between the *Ae. speltoides* chloroplast genome and the chloroplast genome of *T. aestivum*, but these studies were based only on a small region of the chloroplast genome or on RFLP markers. The large 37 kb sequence used in our analysis showed clearly that the *T. aestivum* chloroplast is most closely related to the one of *Ae. speltoides*. In addition to the overall higher level of sequence homology, an abundance of diagnostic nucleotide substitutions demonstrated the close relationship between the *T. aestivum* and *Ae. speltoides* chloroplast sequences.

Additionally, our data allowed us to estimate that the B genome donor diverged from *Ae. speltoides* approximately 780,000–980,000 years ago (again depending on the estimate used). Because *Ae. speltoides* is a strong outbreeder [12], a large number of haplotypes may exist and further sampling of *Ae. speltoides* accessions could lead to the discovery of the same combination of haplotypes that the B genome of *T. aestivum* contains.

### Goatgrass chloroplasts are closely related to those of D genome species

The phylogenetic tree involving the two tetraploid taxa *Ae. geniculata* and *Ae. cylindrica* showed that their chloroplast genomes are closest to that of the D genome species *Ae. tauschii*. Because both, the U and M genomes were placed very near the D genome in previous studies [15], [16], we can not draw any conclusions as to which of the two contributed the chloroplast in the polyploidisation event that led to the formation of *Ae. geniculata*. In contrast, some conclusions are possible for *Ae. cylindrica* which contains the C and D genomes: The Triticeae C genome was described to be more distant from D than both U and M [15]. Thus, our data indicate that the chloroplast of *Ae. cylindrica* was donated by the D genome parent. Previous studies suggested that multiple independent polyploidization events have led to the formation of *Ae. cylindrica* species, with both the C and the D genome donors acting as maternal parents [17]. We therefore conclude that the *Ae. cylindrica* accession (TA 2204 = AE 719) used in this study represents a lineage where the D genome donor was the maternal parent. Furthermore, we can state that the chloroplasts of the D genome and the two goatgrass species diverged only within the past 1.1 to 1.6 Myr. Precise relationships and origins of the individual chloroplast donors, however, can only be determined once chloroplasts from diploid C, U and M genome species are sequenced.

### The advantages and pitfalls of chloroplast dating

Here we used a large part of the chloroplast genome to determine the divergence times of several Triticeae species. Chloroplast sequences have been previously used to determine the divergence time of monocots and dicots [48] as the evolutionary distance between them is relatively large (approximately 130 MYA). There are examples of where whole chloroplast sequences have been used to measure phylogeny and estimate divergence times of closely related species. This was utilized previously [49], where chloroplast genomes from 47 apple taxa were used, including both wild and domesticated taxa. By using the whole chloroplast sequence, a complete phylogeny and estimates of divergence times of the taxa could be obtained [49].

Robust calibration dates are an important consideration when anchoring the tree for the purpose of dating divergence times. Due to the absence of a fossil record in the Triticeae, the calibration dates had to be taken from more distantly related species. We therefore used as calibration dates the divergence of *O. sativa* and *B. distachyon*, which were estimated to have diverged between 40–53 MYA and 32–39 MYA from the Triticeae, respectively. These estimates were based on the comparison between orthologs gene pairs from rice, *Brachypodium* and Triticeae species [23].

**Figure 5:**
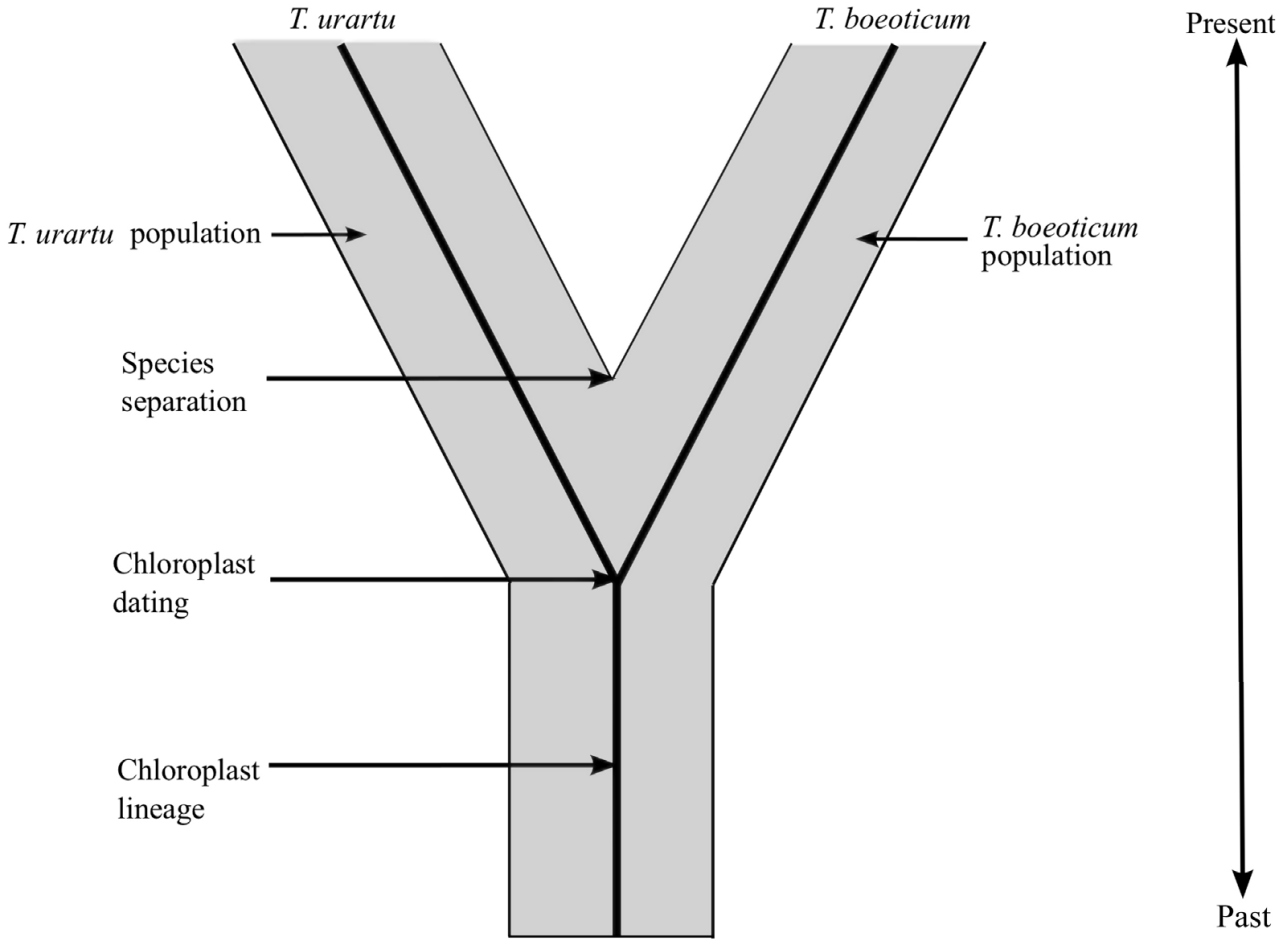
Diagram showing how haplotype divergence and/or incomplete lineage sorting can result in an over-estimation of divergence times, using *T. boeoticum* and *T. urartu* as examples. Haplotypes and/or chloroplast lineages diverge before the formation of the new species (i.e. the complete reproductive isolation of the two populations). Consequently, divergence time estimates derived from sequence data are, in principle, always over-estimates.

The use of chloroplast sequences to estimate species divergence is, in principle, not problematic if the species are separated by a large evolutionary distance. However, with shorter evolutionary times, such as those seen in the Triticeae which are typically less than 10 MYA, the accuracy of estimating the divergence time based on chloroplast genome sequences decreases. The reason is that the possible presence of multiple haplotypes and/or independent chloroplast lineages does not correspond with the actual species divergence. The example in Figure 5 shows that lineages of chloroplast haplotypes which are present in two species today might have diverged substantially earlier than the actual species. Indeed, this is exactly what was found for mitochondrial DNA which was used to estimate the divergence times within a series of bird species: all divergence time estimates were almost double the divergence dates based on the fossil record [50]–[52].

This highlights a principle problem of molecular dating, namely that all divergence times are over-estimated. This is because various ancient haplotype lineages can be present and be recombined within a population or species. Only after species become reproductively isolated, haplotype lineages can no longer mix. Therefore, molecular dating can only estimate the divergence of a particular genetic locus (in our case the chloroplast) but not the divergence of species (Figure 5). Thus, for example the estimated 600,000–700,000 years for the formation of the species boundary between *T. boeoticum* and *T.urartu* has to be seen as the upper limit. This can also explain the discrepancy between our estimated 270,000–280,000 years for the *T. boeoticum*/*T. monococcum* divergence and the 12,000 year-old archaeological evidence: Species have to be morphologically different to be distinguished in the archaeological or fossil record. Thus, archaeological or fossil evidence must always lead to under-estimated divergence times.

In conclusion and with these limitations in mind, we suggest that the use of a large 37 kb region of the chloroplast genome provided robust and relatively precise divergence time estimates for the main Triticeae species. In particular, we were able to describe relationships and divergence times between wheat and its close relatives in great detail. For future studies, it will be highly interesting to include sequences of other wild tetraploid Triticeae species (e.g. *T. dicoccoides* and *T. araraticum*), to place them on the phylogenetic tree and calculate their divergence times.

## Methods

### Chloroplast assembly

Chloroplast assembly was conducted using Newbler at default settings (454 Life Sciences, Roche). The complete 454 read dataset for each species was used for the purposes of *de novo* assemblies. In all cases, the largest contigs produced during the assembly process belonged to the chloroplast. For all taxa, chloroplasts were assembled into very few (3 to maximum 8) large contigs. These contigs were then arranged into the correct order using dot plots against reference chloroplast sequences. The chloroplast of *H. vulgare* (accession number NC008590), downloaded from NCBI, was used as reference for the construction of the chloroplasts from *H. vulgare* cv. Barke and *H. spontaneum* accessions FT11 and FT462. The *T. aestivum* (accession number NC002762) chloroplast sequence downloaded from NCBI was used a reference sequence for the assemblies of *S. cereale* cv. Imperial, *T. urartu* accession EP0471 , *Ae. speltoides* var. *ligustica* accession SPE0061, *Ae. tauschii Coss.* subsp. *strangulata* accession AE429, *T. boeoticum* accession 1628, *T. monococcum* accession 2240, *Ae. cylindrica* accession TA2204, *Ae. geniculata* accession TA1800, and for the re-assembly of *T. aestivum* cv. Chinese Spring.

### Alignments and phylogenetic analyses

Phylogenetic analysis was carried out using a 37 kb region at the start of the LSC. The previously published chloroplast sequences of *B. distachyon* accession number NC011032 and *O. sativa* accession number NC001320 were used as outgroups to anchor the tree. Alignment of all the 37 Kb sequences was done using SeaView [53]. jModeltest [38] was used to obtain the substitution model by implementing the hierarchical likelihood ratio test. According to the Akaike information criterion (AIC) the model best fitting the observed data was GTR+G+I (general time reversible). The maximum likelihood tree was drawn using MEGA 5.0 with 1000 bootstrap replicates.

The model parameters were used in the PAUP* program for the likelihood estimation of the branch lengths of the tree. These branch length estimates of the tree were used to compute the divergence times of all the species from *T. aestivum* using the the semiparametric penalized likelihood method implemented in the r8s program [54], the smoothing parameter was also estimated using the method described by the same authors [54]. *O. sativa* was used as the outgroup to root the tree and the outgroup was pruned before the divergence times were estimated. The divergence time calculations were based on the assumption that *B. distachyon* and *T. aestivum* diverged between 32–39 MYA [22], [23], [55]. 100 Bootstrap replicates were conducted on the dating using fseqboot, which is part of the Phylip package, and r8s boot kit to transform the replicates for use in the r8s program.

### Calibration and estimating divergence times of Triticeae

Due to the absence of good fossil evidence for the Triticeae, calibration of the nodes in the tree was based upon previous molecular data, using the confidence intervals previously stipulated from these findings. Three nodes were selected for the purposes of calibration and these included the divergence times of *O. sativa* 40–53 MYA [23], *B. distachyon* 32–39 MYA [23] and *H. vulgare* 6–15 MYA [26].

Divergence time estimates were estimated using the Bayesian method implemented in the BEAST program [40]. This software was used to infer tree topology, branch lengths and nodal ages using Bayesian inference and Markov Chain Monte Carlo (MCMC) analysis. This was conducted using the whole aligned 37 Kb sequence and a partitioned data set containing 12 genes and 2 intergenic sequences. The genes used in the partitioned dataset include *atpF*, *atpH*, *atpI*, *matK*, *psbA*, *psbC*, *psbD*, *psbK*, *psbZ*, *rpoB*, *rpoC1*, and *rpoC2* with all of these genes being located within the 37 kb region. The intergenic sequences used in the analysis were between the trnS tRNA and the *psb*D gene, which resulted in a sequence of approximately 1080 Bp and the second intergenic sequence is located between the trnC tRNA and the *rpo*B gene with an approximate length of 1140 Bp. The individual gene sequences were aligned and concatenated to produce a total aligned sequence of 19033 Bp. The GTR+G+I substitution model was selected for the genes *atpF*, *atpH*, *atpI*, *psbC*, *psbZ*, *rpoB*, *rpoC1*, and *rpoC2* in the partition, with four gamma categories, with the HKY+G and four gamma categories substitution model was selected for *matK*, *psbA*, *psbD*, *psbK* and for the two intergenic regions, with an uncorrelated relaxed clock model being used, as this allows for rate variation across the branches, and a Yule tree prior was used to model speciation. Two independent MCMC runs were performed for 10,000,000 generations and sampling was conducted every 100th generation. *Brachypodium distachyon* was constrained as the outgroup with a mean of 35 Ma and a standard deviation of 1. Convergence between the runs and the amount of burn in were determined using Tracer 1.5 [40], this was used to assess the effective sample size (ESS) and to check the consistency of the result. TREEANNOTATOR 1.6.2 Drummond2007 was used to calculate a maximum clade probability tree using a posterior probability limit of 0.5, with the final tree being visualised in FIGTREE 1.3.1.

### Data deposition

The chloroplast genomes described in this study were deposited at GenBank under the following accession numbers: *Ae. speltoides*: JQ740834, *Ae. tauschii*: JQ754651, *H. vulgare* (cv. Barke): KC912687, *H. spontaneum* (accession FT11): KC912688, *H. spontaneum* (accession FT462): KC912689, *T. monococcum*: KC912690, *S. cereale*: KC912691, *T. boeoticum*: KC912692, *T. urartu*: KC912693, *T. aestivum*: KC912694, *Ae. cylindrica*: KF534489, *Ae. geniculata*: KF534490.
